# REBOA: is it ready for prime time?

**DOI:** 10.1590/1677-5449.030317

**Published:** 2017

**Authors:** Jay Doucet, Raul Coimbra

**Affiliations:** 1 University of California San Diego – UCSD, Division of Trauma, Surgical Critical Care, Burns and Acute Care Surgery, San Diego, CA, USA.

Death from traumatic injury is the leading cause of loss of productive years of life worldwide, responsible for about 10% of fatalities.^1^ Uncontrolled hemorrhage is responsible for about one third of those trauma deaths. Many of these deaths could be prevented in organized trauma systems by use of extremity bleeding control via methods such as direct pressure, effective hemostatic dressings, and tourniquets, in combination with rapid transport to a trauma center. However, non-compressible torso hemorrhage is difficult to manage in the prehospital phase of care and is now the leading cause of preventable death in organized trauma systems.^2^ Traditionally, these injuries can only be definitively managed in the operating room or angiography suite, without any practical method of pre-procedural hemorrhage control. The window of opportunity for control of non-compressible torso hemorrhage is narrow and delays in management lead to preventable deaths.

Resuscitative endovascular balloon occlusion of the aorta (REBOA) is an endovascular procedure that has recently been advocated for control of uncontrolled torso hemorrhage below the diaphragm. Balloon occlusion of the aorta was advocated by Hughes as early as the Korean War.^3^ Balloon occlusion of the aorta has been used routinely in endovascular management of abdominal aortic aneurysms (EVAR) via large diameter sheaths, typically 12-14Fr or larger. Recent advances, with development of balloon catheters deliverable via 7Fr sheaths, have led to new enthusiasm for the technique for trauma patients.^4^ However, although there is considerable enthusiasm for the new 7Fr catheter REBOA technique that has now become commercially available, the evidence of efficacy is limited.

REBOA is very attractive as a method of hemorrhage control because it can provide total occlusion of the aorta either just above the diaphragm (Zone I), to control intraabdominal bleeding, or above the aorto-iliac bifurcation (Zone III), to control bleeding in the pelvis. The REBOA catheter is inserted via a femoral artery sheath which can be placed via palpation of a pulse, using ultrasound, or by cut-down. It provides an alternative to aortic clamping or compression via a thoracotomy or laparotomy. It can also be performed in non-procedural areas such as the Emergency Room or even in the prehospital environment.

Animal studies have shown that REBOA effectively controls hemorrhage from otherwise lethal injuries, without a need for fluoroscopy for catheter placement.^5^

Clinical data on REBOA are available from two Japanese registry studies^6^,^7^ and 9 small clinical series that have been well summarized by Perkins et al.^8^ The registry studies, by Norii et al. and Inoue et al., showed no overall increase in survival in 452 and 625 REBOA patients compared to propensity score-adjusted untreated patients. There was actually increased mortality in the Norii study (61.8% vs. 45.3%: C.I. 10.9 to 22.0%).^6^ There were no survivors of Zone I REBOA occlusion lasting longer than 45 minutes.^7^

Overall survival in the nine clinical series was 39% percent of 183 patients – survival after Zone I REBOA was 39.4% and survival after Zone III REBOA was 54%.^8^ There was no direct comparator group for these patients receiving REBOA. Comparisons are sometimes made with Emergency Department thoracotomy (EDT) patients with sub-diaphragmatic injuries who typically have overall survival much lower than 10%, but these are not really equivalent as REBOA patients must have some vital signs at presentation and EDT patients usually do not.

There has been some enthusiasm for use of prehospital REBOA performed by trained providers, and there is a case report from the UK of a patient who survived severe pelvic fractures with severe shock after prehospital REBOA and rapid transportation by the London organized trauma system.^9^

The greatest limitation to REBOA is the ischemia caused by total aortic occlusion. Animal studies suggest that Zone I REBOA is survivable for 60 minutes and Zone III for 90 minutes.^4^,^5^ However, the Norii registry study shows that Zone I occlusion for 45 minutes was uniformly lethal and there were only two survivors after 90 minutes of REBOA occlusion in the Inoue registry study.^6^ Once the REBOA catheter is inflated, the time to obtain definitive control of bleeding is limited and the need is absolute. As a result, in the USA REBOA has been performed at major trauma centers that have rapid access to surgical or interventional radiology control as a method to stabilize a patient who needs to be transported between the Emergency Department (ED) and a waiting operating room and/or angiography suite. Delays to definitive control of bleeding are intolerable with REBOA placement.

An additional serious limitation of REBOA is the need for rapid and accurate placement. The steps involved in the procedure using the new 7F REBOA catheter introduced in the USA in 2016 are femoral artery puncture, placement of a 7F arterial sheath, advancement, positioning and inflation of the REBOA catheter, and later deflation and removal of the REBOA balloon after definitive hemorrhage control, followed by removal of the arterial sheath.^10^ The new 7F REBOA catheter does not require use of a long Amplatz-type wire or fluoroscopy. While the procedure is conceptually straightforward, placement of arterial sheaths in severely shocked patients can be challenging and time-consuming even for experienced providers, and gaining arterial access is usually the longest portion of the procedure.

Complications of REBOA are numerous and include death from cardiovascular collapse on balloon deflation due to return of acidotic, hyperkalemic, and hypocalcemic blood from the lower body combined with an abrupt reduction in afterload.^11^ Prolonged ischemia followed by reperfusion results in multiple organ failures including acute kidney injury, liver failure, spinal cord infarction, intestinal ischemia, myonecrosis, limb loss, and death. Vascular complications were common during use of the 12-14F arterial sheath, including improper placement, laceration of vessels, dissections, pseudoaneurysms, and distal limb ischemia and loss. It is hoped that the new 7F sheath and catheter will avoid some vascular complications with its smaller size, however there have been already been vascular and other complications with the new device.

The issue of ischemia-reperfusion injury from complete occlusion of the aorta by REBOA has led to research into “partial-REBOA” or “P-REBOA”.^12^ P-REBOA allows titration of partial deflation of the balloon to allow some distal perfusion while maintaining afterload, possibly by an automated device. Studies have been limited to two animal reports. It is possible that the future of management of non-compressible hemorrhage may involve prehospital imaging and detection and placement of an endovascular device that may provide afterload support, improved proximal perfusion, and oxygenation, and possibly allow distal perfusion or even delivery of agents to reduce distal ischemia-reperfusion injury.^13^

However, current providers must recognize that REBOA lacks evidence better than Level III and involves a considerable risk of complications. REBOA works best within a trauma system in which rapid transport and delivery to definitive bleeding control in the form of an open operating theatre, angiographic suite, or hybrid room are available. The extra time garnered by current REBOA technology is very short. It should also be remembered that the time for arterial access and deployment of REBOA may in fact take longer than rapid intervention protocols, such as direct-to-operating room (OR) admission of patients identified by Emergency Medical Services as possibly having non-compressible torso hemorrhage, bypassing the ED. In our center, having a direct-to-OR resuscitation strategy reduced time-to-incision by 21-64 minutes, which may eliminate the need for ED REBOA placement.^14^

Unfortunately, in the USA the new 7F REBOA technology has been marketed to many types of providers without always distinguishing the type of facility or trauma system in which they work. We believe that REBOA should be considered an investigational technique that needs proper protocols, outcomes analysis, and reporting within a highly organized and collaborative trauma system. There are multicenter consortia in the US and UK accepting REBOA cases for their databases, but the current US FDA device approval does not require such participation by centers using REBOA. Increasingly widespread and unreported use of REBOA without better evidence for safety and efficacy or reporting to national databases may already be upon us.
